# Comparative RNA-seq analysis of transcriptome dynamics during petal development in *Rosa chinensis*

**DOI:** 10.1038/srep43382

**Published:** 2017-02-22

**Authors:** Yu Han, Huihua Wan, Tangren Cheng, Jia Wang, Weiru Yang, Huitang Pan, Qixiang Zhang

**Affiliations:** 1 Beijing Key Laboratory of Ornamental Plants Germplasm Innovation & Molecular Breeding, National Engineering Research Center for Floriculture, Beijing Laboratory of Urban and Rural Ecological Environment, Key Laboratory of Genetics and Breeding in Forest Trees and Ornamental Plants of Ministry of Education, School of Landscape Architecture, Beijing Forestry University, Beijing, 100083, China

## Abstract

The developmental process that produces the ornate petals of the China rose (*Rosa chinensis*) is complex and is thought to depend on the balanced expression of a functionally diverse array of genes; however, the molecular basis of rose petal development is largely unknown. Here, petal growth of the *R. chinensis* cultivar ‘Old Blush’ was divided into four developmental stages, and RNA-seq technology was used to analyse the dynamic changes in transcription that occur as development progresses. In total, 598 million clean reads and 61,456 successfully annotated unigenes were obtained. Differentially expressed gene (DEG) analysis comparing the transcriptomes of the developmental stages resulted in the identification of several potential candidate genes involved in petal development. DEGs involved in anthocyanin biosynthesis, petal expansion, and phytohormone pathways were considered in depth, in addition to several candidate transcription factors. These results lay a foundation for future studies on the regulatory mechanisms underlying rose petal development and may be used in molecular breeding programs aimed at generating ornamental rose lines with desirable traits.

The China rose (*Rosa chinensis*) is widely cultivated around the world and has a high ornamental value. ‘Old Blush’ is an easily propagated diploid cultivar of *R. chinensis*, which has recurrent flowering, double flowers, pink petals, and a pleasant fragrance. ‘Old Blush’ originated in China over a thousand years ago^1^. Its relatively simple genome makes it an ideal subject for molecular biology and cross breeding studies. One study investigated the transcriptome of various tissues of ‘Old Blush’ following exposure to biotic and abiotic stresses, providing a useful resource for genetic mapping and sequencing of the rose genome^1^. Another study analysed expression of the scent-related genes during flowering in ‘Pallida’, which is closely related to ‘Old Blush’^2^. The main ornamental features of *R. chinensis* are its petals. Petals are exclusively floral organs, and as such have some specialized biological processes that may require specific signal transduction mechanisms. Many studies have examined the regulation of plant growth and development in model plant *Arabidopsis thaliana*^3^; however, research into *R. chinensis* flowering is limited.

The ornamental quality of a rose largely depends on the size and pigmentation of its petals. The phenotype of rose petals changes during development; in ‘Old Blush’, the process begins with the formation of small, green petals that undergo an early growth phase driven mainly by cell division and accompanied by an initiation of pigment synthesis. The petal cells grow slowly and their pigment contents rapidly increase until they reach maximum pigmentation, resulting in pink petal colouration. Later, petal growth occurs predominantly by cell expansion, allowing the petal to reach its final size. The genes involved in anthocyanin biosynthesis and petal expansion have been described^4^,^5^,^6^, and numerous studies have focussed on the regulation of senescence in cut roses^7^,^8^,^9^. However, the regulatory mechanisms controlling rose petal development are poorly understood.

Rose petal growth and development depend on the phytohormone-mediated regulation of gene expression via transcription factors^10^; however, little is known about the processes involved. RNA-seq technology enables the analysis of whole transcriptomes. By detecting changes in unigene expression, this technology generates large-scale gene expression datasets that can guide future research. In this study, we used RNA-seq technology to analyse the transcriptome of ‘Old Blush’ petals throughout their development, to gain insight into the processes underlying rose petal development at the transcriptional level.

## Result

### Analysis of ornamental characteristics in developing *Rosa chinensis* petals

**Figure 1:**
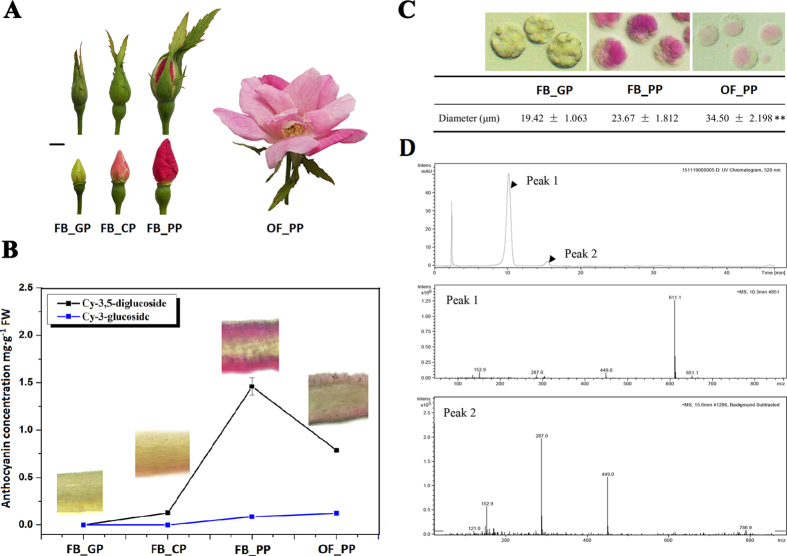
The definition and characterisation of RNA-seq experimental materials in *Rosa chinensis* ‘Old Blush’. (**A**) The four stages of petal development: FB_GP, green petals in the flower bud; FB_CP, colour-changing petals in the flower bud; FB_PP, pink petals in the flower bud; OF_PP, pink petals of the open flower. Plant materials grown under a 12 h light (6:00 am–18: 00 pm)/12 h dark (18:00 pm–6:00 am) photoperiod and a 25 °C day/18 °C night temperature regime in the greenhouse. Petals have been collected at 12:00 am. Scale bar = 5 mm. (**B**) The anthocyanin concentration in the petals of the four developmental stages. Cy-3,5-diglucoside (black line) is the main anthocyanin in petals of ‘Old Blush’. Cy-3-glucoside (blue line) is the other detectable anthocyanin. The inset images display a longitudinal cross-section of the petals at each stage of development. The error bars represent standard errors. (**C**) Petal protoplasts at stages FB_GP, FB_PP, and OF_PP and their respective mean protoplast diameter. Values represent the means and SD of three biological replicates. Asterisks denote significant differences compared with the diameter of stage FB_GP at P < 0.01 (Student’s t-test). (**D**) LC-MS chromatograms of Rosa chinensis ‘Old Blush’ petals at stage FB_PP. Absorbance was measured at 520 nm. Peak 1: Cy-3,5-diglucoside, m/z = 611.1; Peak 2: Cy-3-glucoside, m/z = 287.0 and 449.0.

The petals of developing ‘Old Blush’ flowers were divided into four typical stages: green petals in the young flower bud (FB_GP), colour-changing petals in the flower bud (FB_CP), pink petals in the flower bud (FB_PP), and pink petals in the open flower (OF_PP; Fig. 1A). Initially, the FB_GP petals were small, thin and tightly stacked together. Then, the lower epidermis of the petals began to produce pigment (FB_CP; Fig. 1B). Pigment accumulation increased rapidly after FB_CP and peaked at stage FB_PP. Finally, the petals expanded and the pigmentation was distributed, resulting in a large pink bloom. High-performance lipid chromatography (HPLC) and liquid chromatography/mass spectrometry (LC-MS) analysis revealed two major anthocyanins in the petals: cyanidin-(Cy-) 3,5-diglucoside and Cy-3-glucoside (Fig. 1B and D). The concentration of the most abundant of the two, Cy-3,5-diglucoside, peaked at 1.46 ± 0.089 mg · g^−1^ FW in stage FB_PP, while the Cy-3-glucoside concentration peaked in stage OF_PP at 0.12 ± 0.0015 mg · g^−1^ FW (Fig. 1B). The isolated petal protoplasts of FB_PP petals were slightly larger than those of FB_GP, and the vacuoles of the former were a darker shade of pink (Fig. 1C), corresponding to their high concentration of anthocyanin pigments. Both FB_GP and FB_PP protoplasts contained several small vacuoles, which contrasted with the single, larger vacuole observed in the petal protoplasts of OF_PP.

### High-quality Illumina sequencing reveals the transcriptome of developing rose petals

RNA isolated from the petals of the four developmental stages was sequenced using the Illumina platform, generating 598 million high-quality reads representing 89.72 Gbp, the error rate of sequenced bases in all samples were not more than 0.02% (Supplementary Table S1). As *R. chinensis* did not have an appropriate reference genome sequence, the Trinity method^11^ was used to *de novo* assemble all clean reads. The length frequency distribution and the mean lengths of assembled transcripts and unigenes are displayed in Supplementary Tables S2 and S3. A uniform distribution of transcripts was detected across the reference sequences in all samples (Supplementary Figure S2). For each of the four sampling stages, the Pearson correlation coefficients (R) between biological replicates were high (R^2^ > 0.88 in all cases, Supplementary Figure S3), indicating a good repeatability and are liable dataset. Over 77% of the reads could be mapped back to the assembled transcripts in the eight samples (Supplementary Table S4).

Seven databases (Nr, Nt, Pfam, KOG, Swiss-prot, KO and GO) were used to annotate all unigenes with comprehensive gene function information (Supplementary Table S5). A total of 61,456 unigenes were successfully annotated using at least one database, with 40.3% (45,138) of unigenes annotated using Nr and 32.0% (35,830) using GO. The ‘Old Blush’ petal unigenes were relatively similar to those of *Fragaria vesca*, with 54% of sequences showing homology by BLAST analysis (Supplementary Figure S4). The rose petal unigenes were analysed for predicted function and gene classification using the GO, KOG, and KO databases. The unigenes were annotated with 55GO terms (Supplementary Table S6), the most common of which were the biological process categories ‘cellular process’ (19,558 unigenes) and ‘metabolic process’ (18,860). The ‘single-organism process’ category defines a biological process involving only one organism, and the 14,661 *R. chinensis* unigenes annotated as such may be related to flowering organisms specifically. There were 2,090 unigenes annotated as ‘signal transduction mechanisms’ based on the KOG database, and the most frequent category was ‘General function prediction only’ (3,389) (Supplementary Table S7). The KO database identified the putative biological pathways of the unigenes (Supplementary Table S8), with 2,229 unigenes attributed to the ‘signal transduction’ pathway; this count was similar to the number identified by the GO- and KOG-based analyses.

### Dynamic comparative analysis of differentially expressed genes (DEGs) during petal development

**Figure 2:**
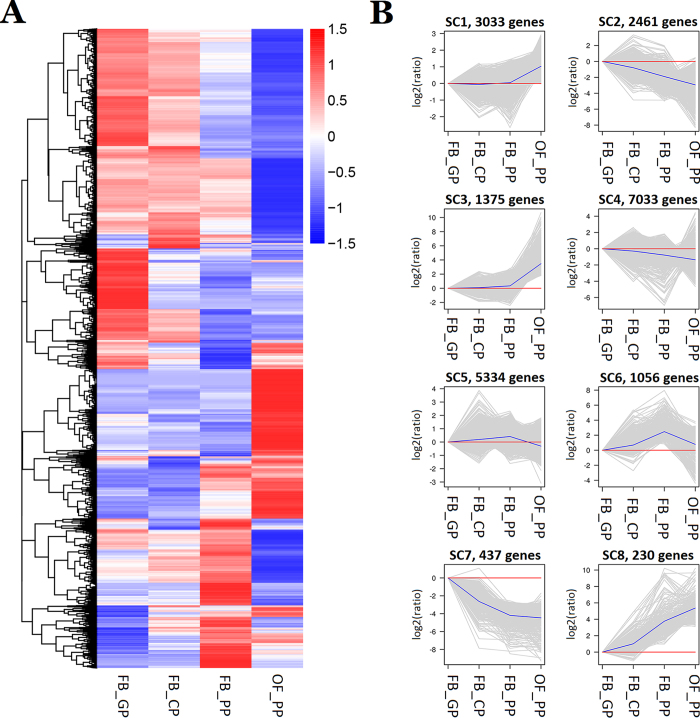
Analysis of differentially expressed genes (DEGs) in the four stages of petal development in *Rosa chinensis*. (**A**) Overall cluster analysis of DEGs in four stages (FB_GP, FB_CP, FB_PP, and OF_PP). FPKM (fragments per kilo base of transcript per million base pairs sequenced) was used to estimate the level of gene expression. The colour change from red (highly expressed) to blue (low expression) represents the relative expression level value log2 (ratios). (**B**) The H_cluster analysis of DEGs in FB_GP, FB_CP, FB_PP, and OF_PP resulted in their categorisation into eight expression pattern types, SC1-SC8. SC1 and SC3: the cluster of genes that were upregulated by a log2 ratio of about one or four from FB_PP to OF_PP. SC2 and SC4: the cluster of genes which average log2 ratio of about negative three or one from FB_GP to OF_PP. SC5 and SC6: the cluster of genes that were up-regulated from FB_GP to FB_PP and down-regulated from FB_PP to OF_PP. The average log2 ratio was less than one in SC5 and more than one in SC6. SC7 and SC8 present the cluster of genes that were down-regulated or up-regulated more than a log2 ratio of four from FB_GP to OF_PP. The mean expression levels of all genes in each of the eight categories (blue line) are displayed relative to a log2 ratio of 0 (red line). The numbers of DEGs in each cluster are displayed.

RSEM (RNA-Seq by Expectation Maximization) software was used to identify the DEGs between the four petal developmental growth stages (Padj < 0.05). FPKM (fragments per kilo base of transcript per million base pairs sequenced) was used to estimate the level of gene expression. The clustered patterns of all DEGs expression during petal development (Fig. 2A) were created based on their relative expression level value log2 (ratios) (Supplementary Table S9)^12^. Another H_cluster analysis split the DEGs into eight clusters, SC1-SC8, with distinct expression patterns (Fig. 2B). The SC1 and SC3 clusters included genes that were up-regulated by a log2 ratio of one or four, respectively, from FB_PP to OF_PP. The SC2 and SC4 clusters included genes that were down-regulated by a log2 ratio of about three or one, respectively, from FB_GP to OF_PP. The SC5 cluster of genes was up-regulated from FB_GP to FB_PP and down-regulated from FB_PP to OF_PP, and the average range was less than one log2 ratio, whereas the SC6 cluster included genes that up-regulated from FB_GP to FB_PP and down-regulated from FB_PP to OF_PP in expression by more than one log2 ratio. The SC7 and SC8 clusters included genes that were always down-regulated or up-regulated by log2 ratio of more than four from FB_GP to OF_PP. The largest cluster, SC4, contained 7,033 genes that were down-regulated as petal development progressed, while the SC7 cluster contained the 437 genes that were most strongly down-regulated during the process (Fig. 2B). The SC8 cluster comprised the 230 genes most highly up-regulated during the four stages of petal development.

**Figure 3:**
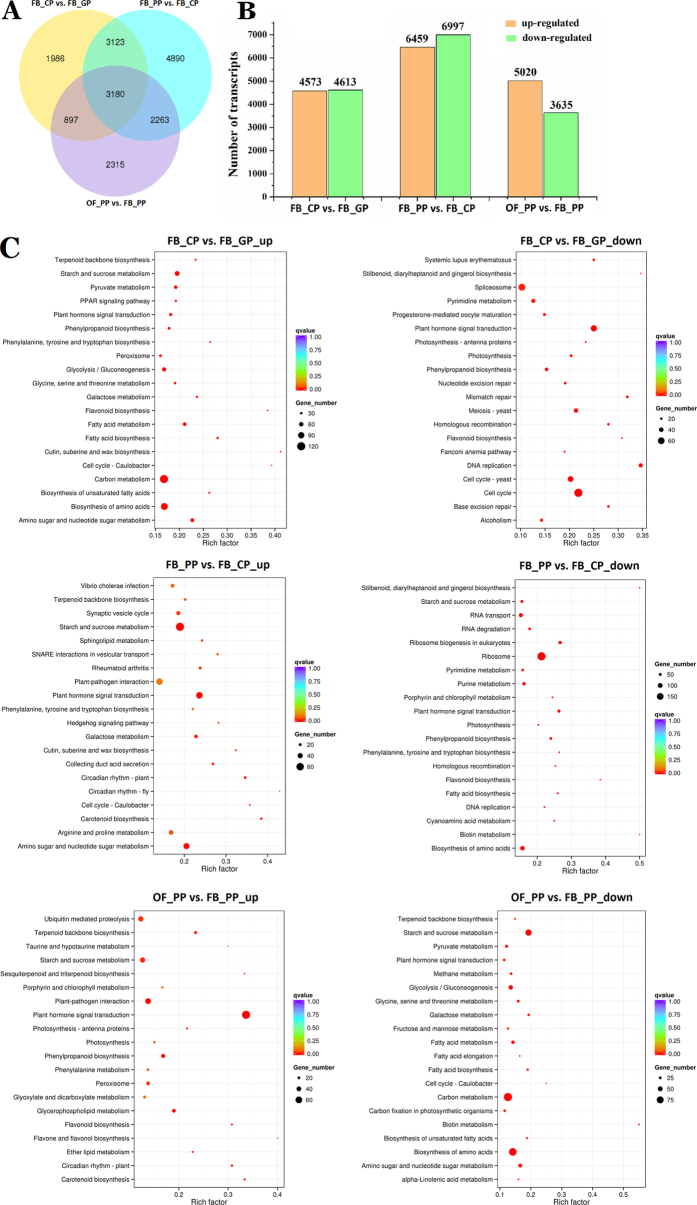
Comparative analysis of differentially expressed genes (DEGs) between the four stages of petal development. (**A**) Venn diagram of the number of DEGs (Padj < 0.05) between the stage comparisons: FB_CP vs. FB_GP, FB_PP vs. FB_CP, and OF_PP vs. FB_PP. (**B**) The number of up-regulated and down-regulated DEGs between the three comparisons (FB_CP vs. FB_GP, FB_PP vs. FB_CP, OF_PP vs. FB_PP). (**C**) KO category enrichment of up-regulated and down-regulated DEGs. The number of genes in each category is equal to the dot size. The dot colour represents the q-value.

We conducted a detailed comparative analysis of the DEGs using three combinations of stages that represented major changes in the petal phenotype (FB_CP vs. FB_GP, FB_PP vs. FB_CP and OF_PP vs. FB_PP). FB_CP vs. FB_GP represented the process which green petals undergo an early growth phase driven mainly by cell division until initiated of pigment synthesis. FB_PP vs. FB_CP represented the process which petal cells grown slowly and their pigment contents rapidly increased until they reached maximum pigmentation. FB_PP vs. FB_CP represented the process which petal growth occurred predominantly by cell expansion until flower opening. There were 1,986, 4,890, and 2,315 DEGs, respectively, specific to these three comparisons (Fig. 3A, Supplementary Table S10). Between FB_GP and FB_CP, 4,573 and 4,613 transcripts were up- and down-regulated, respectively, while in the FB_PP vs. FB_CP comparison, there were 6,459 and 6,997 transcripts up- and down-regulated, respectively (Fig. 3B). There were more up-regulated transcripts (5,020) than down-regulated (3,635) in the OF_PP vs. FB_PP comparison, indicating that during floral opening, the number of DEGs decreased and the down-regulated genes were relatively reduced.

An analysis of the DEGs from the three comparisons using the GO database revealed that 20 categories of biological processes (Supplementary Figure S5) were enriched in the up- and down-regulated DEGs, except in the down-regulated DEGs of FB_CP vs. FB_GP, where only four terms were enriched. There were only two terms that were enriched in the up-regulated genes of FB_CP vs. FB_GP, but 19 terms enriched in the down-regulated genes. Interestingly, the ‘cellular component’ category showed significant changes in all analysis groups; however, when the flower opened, the down-regulated DEGs were not enriched in this category. These data suggest that complex gene regulatory mechanisms underlie petal development.

The KEGG Orthology-Based Annotation System (KOBAS) was used to perform a further functional classification and pathway assignment of the up- and down-regulated DEGs (Fig. 3C). The pathways enriched in the DEGs that were up-regulated in FB_CP vs. FB_GP were ‘carbon metabolism’ and ‘biosynthesis of amino acids’, while in contrast, these pathways were enriched in the down-regulated DEGs of OF_PP vs. FB_PP. ‘Plant hormone signal transduction’ was the most enriched pathway in the up-regulated DEGs of OF_PP vs. FB_PP, which was also enriched in the up-regulated DEGs of FB_PP vs. FB_CP but contrastingly enriched in the down-regulated DEGs of FB_CP vs. FB_GP, suggesting a potentially important role for phytohormone signalling in rose petal development.

### Identification of genes involved in the anthocyanin biosynthesis pathway and petal expansion

**Figure 4:**
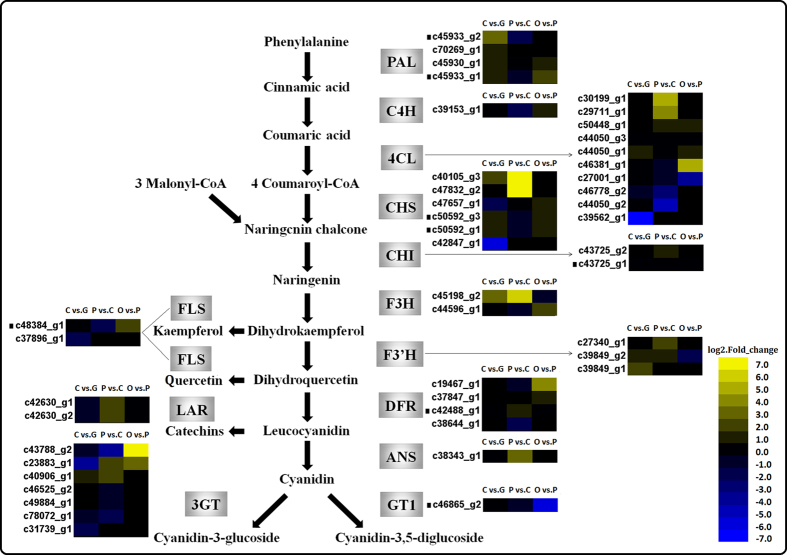
Heat maps of the expression of anthocyanin biosynthesis pathway genes during *Rosa chinensis* ‘Old Blush’ petal development. “C vs. G” represents FB_CP vs. FB_GP, “P vs. C” represents FB_PP vs. FB_CP, and “O vs. P” represents OF_PP vs. FB_PP. Yellow and blue represent up- and down-regulated transcripts, respectively, from the three comparisons (log2-fold change), while a black dot indicates a transcript for which the putative homolog gene has been previously registered in the NCBI database. PAL: phenylalanine ammonialyase; 4CL: 4-coumarate-CoA ligase; C4H: cinnamate-4-hydroxylase; CHS: chalcone synthase; CHI: chalcone isomerase; F3H: flavanone 3-hydroxylase; F3′H: flavonoid 3′-hydroxylase; DFR: dihydroflavonol 4-reductase; ANS: anthocyanidin synthase; GT1: anthocyanidin 5, 3-O-glucosyltransferase; 3GT: anthocyanidin 3-O-glucosyltransferase; FLS: flavonol synthase; LAR: leucoanthocyanidin reductase. All genes are listed in detail in Supplementary Table S11.

To elucidate the genetic regulation of rose petal pigmentation, the genes were filtered for those believed to be involved in the putative anthocyanin biosynthesis pathway for *R. chinensis* ‘Old Blush’ (Fig. 4, Supplementary Table S11), which was determined from previous research. The functions of most of the enzymes in the anthocyanin biosynthesis pathway have been previously reported^4^. In FB_PP vs. FB_CP, the expression of several putative anthocyanin biosynthesis genes was up-regulated, including those encoding 4CL (unigenes c30199_g1 and c29711_g1), CHS (c40105_g3 and c47832_g2), CHI (c43725_g2), F3H (c45198_g2), F3′H (c27340_g1 and c39849_g2), DFR (c42488_g1), ANS (c38343_g1), LAR (c42630_g1 and c42630_g2), and 3GT (c23883_g1 and c40906_g1). The CHS-annotated unigenes had more than a seven-fold up-regulation in expression in FB_PP vs. FB_CP.

**Figure 5:**
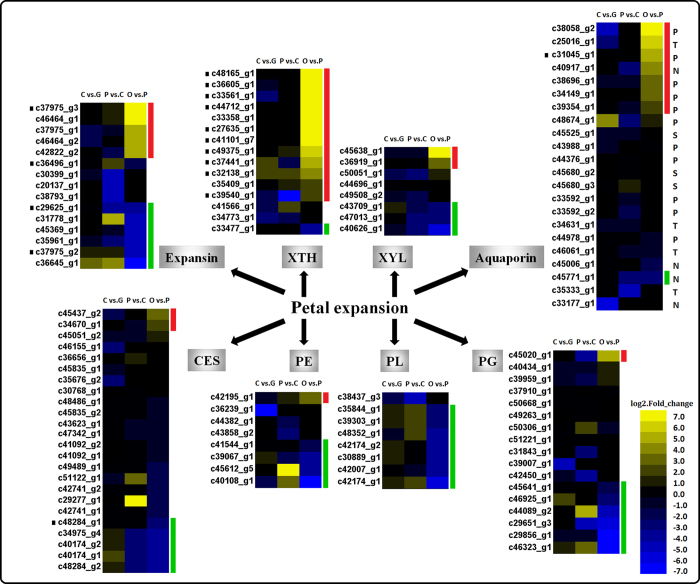
Heat maps of petal expansion-related genes during *Rosa chinensis* ‘Old Blush’ petal development. “C vs. G” represents FB_CP vs. FB_GP, “P vs. C” represents FB_PP vs. FB_CP, and “O vs. P” represents OF_PP vs. FB_PP. Yellow and blue indicate up- and down-regulated transcripts, respectively, from the three comparisons (log2-fold change), while a black dot indicates a transcript for which the putative homolog gene has been previously registered in the NCBI database. XTH: xyloglucan endotransglucosylase/hydrolase; XYL: xylosidase; CES: cellulose synthase; PE: pectinesterase; PG: polygalacturonase; PL: pectate lyase. P: plasma membrane intrinsic protein (aquaporin); T: tonoplast intrinsic protein (aquaporin); S: small basic intrinsic protein (aquaporin); N: nodulin-26-like intrinsic membrane protein (aquaporin). Red and green lines indicate the genes with a log2-fold change of >2. All genes are listed in Supplementary Table S11.

The expansin family of proteins have previously been reported to be involved in the disruption of the non-covalent bonds between the cellulose microfibrils and the cross-linking glycans of the cell wall, thereby promoting ‘wall creep’^13^. Fifteen putative members of the expansin family, including 14 α-expansins and one β-expansin, were determined in the *R. chinensis* samples (Fig. 5, Supplementary Table S11). Among them, five α-expansins (c37975_g3, c46464_g1, c37975_g1, c46464_g2, c42822_g2) had more than a five-fold increase in expression in stage OF_PP vs. FB_PP, which indicates an up-regulation during the period of rapid petal expansion. Conversely, five α-expansins (c29625_g1, c31778_g1, c45369_g1, c37975_g2, c36645_g1) and the β-expansin (c35961_g1) were markedly down-regulated in this stage. Xyloglucan endotransglycosylase/hydrolase (XTH) has also been shown to participate in cell expansion, loosening and rearranging the cell wall fibers in growing tissues^14^,^15^. Fifteen unigenes were annotated as XTH in the *R. chinensis* transcriptome, the expression of 13 of which was highly up-regulated in OF_PP vs. FB_PP. Aquaporins facilitate the passage of water and/or small neutral solute fluxes across membranes and play crucial roles in plant growth^16^. A total of 22 aquaporins were annotated in the petal transcriptome, including eleven plasma membrane intrinsic proteins (PIPs), four tonoplast intrinsic proteins (TIPs), four nodulin-26-like intrinsic membrane proteins (NIPs) and three small basic intrinsic proteins (SIPs) (Fig. 5, Supplementary Table S11). The expression of five PIPs (c38058_g2, c31045_g1, c38696_g1, c34149_g1 and c39354_g1), one TIP (c25016_g1), and one NIP (c40917_g1) was almost twice as high in OF_PP than in FB_PP. The expression levels of most SIPs did not change during petal development. The expression of unigenes annotated as genes involved in cell wall synthesis, modification or hydrolysis, such as cellulose synthase (CES), xylosidase (XYL), pectin esterase (PE), polygalacturonase (PG), and pectate lyase (PL)^17^,^18^,^19^,^20^, were also assessed, as these may participate in rose petal expansion (Fig 5, Supplementary Table S11). The expression of 10 CESs, three XYLs, four PEs, seven PLs, and six PGs in OF_PP petals was more than twice as high as those in the FB_PP stage, with particularly marked differences in the expression of the PLs.

### Transcriptome analysis of phytohormone pathway genes during petal development

**Figure 6:**
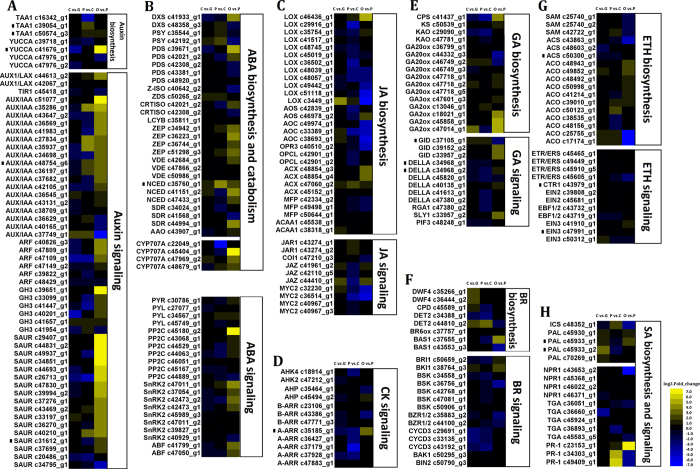
Heat maps of phytohormone-related genes during *Rosa chinensis* petal development. “C vs. G” represents FB_CP vs. FB_GP, “P vs. C” represents FB_PP vs. FB_CP, and “O vs. P” represents OF_PP vs. FB_PP. Yellow and blue indicate up- and down-regulated transcripts, respectively, from the three comparisons (log2-fold change), while a black dot indicates a transcript for which the putative homolog gene has been previously registered in the NCBI database. All genes are listed in Supplementary Table S12. (**A**–**H**) The heat map of major genes involved in the biosynthesis and signalling pathways of (**A**) auxin, (**B**) abscisic acid, (**C**) jasmonic acid, (**D**) cytokinin, (**E**) gibberellic acid, (**F**) brassinosteroid, (**G**) ethylene, and (**H**) salicylic acid.

Plant growth is regulated by hormones such as auxin (AUX), abscisic acid (ABA), jasmonic acid (JA), cytokinin (CK), gibberellin (GA), brassinosteroid (BR), ethylene (ETH) and salicylic acid (SA)^21^, with exogenous AUX, ABA, GA and ETH known to affect petal expansion and flower opening^10^. *R. chinensis* petal unigenes annotated as being involved in known phytohormone biosynthesis and signalling pathways are displayed in Supplementary Table S12, with DEGs during petal development presented in Fig. 6.

Fifty of the petal unigenes were annotated as being part of the previously described AUX signalling pathway^22^. The majority of them had higher expression in OF_PP vs. FB_PP, with particularly high levels of expression in AUX/IAA (c51077_g1, c35286_g1), GH3 (c39651_g1), and SAUR (from c29407_g1 to c39994_g2), which had more than a five-fold degree of up-regulation during this stage. The expression of TAA1 and YUCCA did not alter actively during petal development, except for unigene c41676_g1, which was up-regulated more than eight fold in OF_PP vs. FB_PP. The expression of many ABA biosynthesis genes was up-regulated in OF_PP vs. FB_PP (Fig. 6B), such as those encoding zeaxanthin epoxidase (ZEP), 9-cis-epoxycarotenoid dioxygenase (NCED), short-chain dehydrogenase/reductase (SDR), and abscisic-aldehyde oxidase-like (AAO). The expression of the unigenes (c45404_g1, c47969_g2, c48679_g1) annotated as abscisic acid 8’-hydroxylase (CYP707A), the ABA-catabolism enzyme, was also up-regulated in this stage, suggesting that the balance of ABA biosynthesis and catabolism is tightly controlled during rose petal expansion.

Unigenes annotated as being involved in the GA biosynthesis or signalling pathway were also differentially expressed in OF_PP vs. FB_PP. GA biosynthesis genes encoding an ent-Copalyl diphosphate synthase (CPS; c41437_g1) and three gibberellin 2-beta-dioxygenases (GA2ox; c18021_g1, c45858_g1, c47014_g1) showed a four-fold upregulation in expression, while two gibberellin receptors (GID; c39152_g2, c33957_g2) and SLEEPY1 (SLY1; c33957_g2) from the GA signalling pathway had a three-fold increase in expression, suggesting a role for GA in rose petal expansion. Many unigenes in the JA biosynthesis pathway had a four-fold down-regulation of expression in OF_PP vs. FB_PP, such as those encoding lipoxygenase (LOX; c51118_g1, c3449_g1), allene oxide cyclase (AOC; c49974_g1, c33389_g1, c38693_g1), and 12-oxophytodienoate reductase 3-like (OPR3; c40510_g2). Other important phytohormones had transcriptome level changes during petal development. The two component response regulator ARR-A (A-ARR) encoding gene was down-regulated in FB_CP vs. FB_GP and FB_PP vs. FB_CP. The transcription of ETH, SA and BR pathway genes also have some change during petal development.

### Transcriptomic analysis of transcription factors during petal development

**Figure 7:**
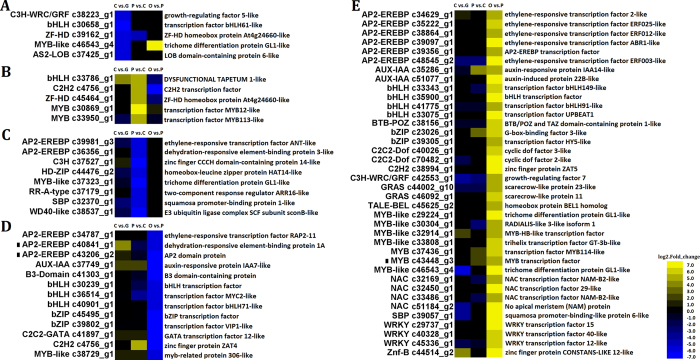
Heat maps of transcription factors (TFs) with significant expression changes during *Rosa chinensis* petal development. “C vs. G” represents FB_CP vs. FB_GP, “P vs. C” represents FB_PP vs. FB_CP, and “O vs. P” represents OF_PP vs. FB_PP. Yellow and blue indicate up- and down-regulated transcripts, respectively, from the three comparisons (log2-fold change), while a black dot indicates a transcript for which the putative homolog gene has been previously registered in the NCBI database. Each gene prediction has been marked on the right. All genes are listed in detail in Supplementary Table S13. (**A**) TFs with ≥ five-fold down-regulated expression in FB_CP vs. FB_GP. (**B**) TFs with ≥ five-fold up-regulated expression in FB_PP vs. FB_CP. (**C**) TFs with ≥ five-fold down-regulated expression in FB_PP vs. FB_CP. (**D**) TFs with ≥ five-fold down-regulated expression in OF_PP vs. FB_PP. (**E**) TFs with ≥ five-fold up-regulated expression in OF_PP vs. FB_PP.

Numerous TFs in the rose petal transcriptome had more than a five-fold change in expression during development, which was the threshold change required to be included in this analysis. In the three comparisons, 69 TFs were selected as potentially being involved in petal development based on their annotations and further confirmation using PlantTFcat^23^ (Fig. 7, Supplementary Table S13). In FB_CP vs. FB_GP, none of the TF genes were up-regulated more than the five-fold threshold, but there were five down-regulated TFs: c38223_g1, c30658_g1, c39162_g1, c46543_g4 and c37425_g1. In FB_PP vs. FB_CP, there were five up-regulated TFs and eight down-regulated TFs that surpassed the threshold. There were 38 up-regulated TFs and 13 down-regulated TFs that surpassed the threshold in the progression from FB_PP to OF_PP. Among these TFs, seven MYBs were up-regulated (c29224_g1 to c46543_g4 in Supplementary Table S13) and one MYB was down-regulated (c38729_g1). The AP2/EREBP TF family is divided into four subfamilies; AP2, DREB, ERF, and RAV^24^. Of the 11 AP2-EREBP TFs in the three stage comparisons, two had more than a five-fold down-regulation in expression in FB_CP vs. FB_GP, while three were down-regulated more than five-fold in FB_PP vs. FB_CP. By contrast, the expression levels of six AP2-EREBP TFs were more than five times greater in OF_PP than FB_PP. The expression of two DREB TFs, c36356_g1 and c40841_g1, was up-regulated more than five-fold in OF_PP vs. FB_PP. In OF_PP vs. FB_PP, the expression of four NAC family TFs was up-regulated dramatically. Unigenes c32169_g1, c33486_g1 and c51184_g2 were annotated as NAM (No Apical Meristem), a NAC gene involved in the development of the shoot apical meristem. Two bZIP TFs were down-regulated more than five-fold in OF_PP vs. FB_PP, while two bZIP TFs were up-regulated past the threshold in this same comparison.

## Discussion

**Figure 8:**
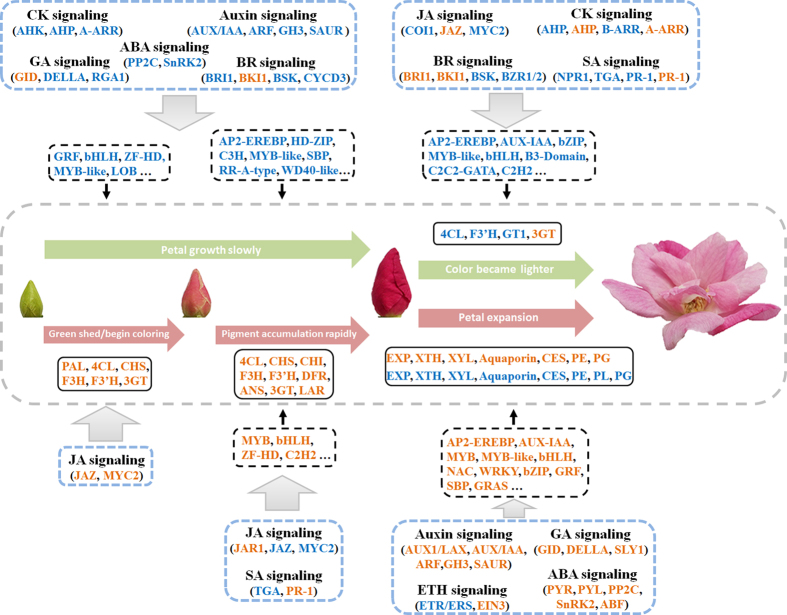
Summary of transcription-level regulation of ‘Old Blush’ petal development. Differentially expressed genes (DEGs) that may regulate petal development include petal colour genes and expansion-related genes (those with more than a one-fold change in expression, Padj < 0.05; box with solid black line), transcription factors (those with more than a five-fold change in expression, Padj < 0.05; box with black dotted line), and phytohormone synthesis and signalling genes (those with more than a one-fold change in expression, Padj < 0.05; box with blue dotted line). These genes were either up-regulated (orange) or down-regulated (blue) as petal development progressed.

Pink roses symbolise warmth and romance. Here, we found that Cy-3,5-diglucoside is the main pigment in the pink petals of the China rose ‘Old Blush’, with several small pigment containing vacuoles in the immature petals combining into a single larger vacuole during petal expansion. Each of the four petal developmental stages had a distinct phenotype, and the DEGs involved petal colour, expansion and phytohormone signalling pathway (log2-fold change ≥ one-fold, Padj < 0.05), TFs (log2-fold change ≥ five-fold, Padj < 0.05) have been collected and summarised in Fig. 8, corresponding to the possible involved biological processes. We detected the expression of 38 genes during petal development using qPCR, which standard errors within reasonable ranges, and the results showed significant specificity as the RNA-Seq data (Supplementary Figure S6, Supplementary Table S14). Many of the unigenes categorised as being involved in ‘cellular component’ pathways were down-regulated in FB_CP vs. FB_GP and FB_PP vs. FB_CP, while in OF_PP vs. FB_PP the majority of both the up- and down-regulated genes were annotated as either ‘metabolic process’ or ‘catalytic activity’. Under the KEGG categories, unigenes annotated as being involved in ‘carbon metabolism’, ‘biosynthesis of amino acids’, and ‘starch and sucrose metabolism’ were the most commonly up-regulated in the FB_CP vs. FB_GP comparison, and were commonly down-regulated in OF_PP vs. FB_PP. Though there were many differences in the transcriptomes of the different developmental stages, the ‘plant hormone signal transduction’ pathways were differently expressed in all of the transcriptome comparisons between stages. These findings suggest that during petal development, complex regulatory systems may exist to control the changes at each stage and to ensure a properly developed bloom.

Flower colour and shape are the most important ornamental characters of *R. chinensis*. Our analysis of the anthocyanin biosynthesis and petal expansion pathway genes (Figs 4 and 5), which focussed on genes with functions known to correspond to the phenotype, such as CHS, F3H, F3′H, DFR, ANS, EXP, XTH, XYL and aquaporin, revealed these pathways to be up-regulated during petal pigmentation or expansion. The unigene of the Cy-3,5-diglucoside catalysing enzyme in *R. hybrida* (RhGT1)^5^, c46862_g2, was not up-regulated but was strongly expressed during the early developmental stages (FB_GP and FB_CP), and decreased in the mature flowering stage (OF_PP). The down-regulated catabolic enzyme GT1 may participate in the later lightening of petal colour by reducing Cy-3,5-diglucoside production in the petals. Previous research has revealed that the enzymatic competition between FLS and DFR determined the balance of flavonols and anthocyanins in petunia, with a disequilibrium of FLS and DFR expression being tightly associated with the formation of white or red coloured flowers^25^. The expression changes of FLS and DFR in ‘Old Blush’ petals were not obvious in the present study; we did not obtain the F3′5′H transcript in this transcriptome, but the down-regulated unigenes from PAL to DFR in the pathway, as well as the single copy of the ANS unigene (c38343_g1), may be the reasons why no pelargonidin or delphinidin was detected in the ‘Old Blush’ petals.

The β-expansins may disrupt different cell wall polymers than the α-expansins^26^; however, the function of β-expansin in petal development was not clear. The expression of the β-expansin unigene detected in the present study, c35961_g1, decreased as petal development progressed, suggesting that it may be involved in this biological process. The large numbers of expansin family members may allow the development of substrate specificity or pH dependence in the enzymes, enabling different expression patterns or upstream signalling, as well as providing some genetic redundancy^27^, and our results presented the possibility that the expansins perform various functions during rose petal development. The unigene c31045_g1 was up-regulated in the open flower. This gene was annotated as RhPIP2;1, which was previously shown to have the same expression pattern as reported previously during petal expansion^28^. It is not clear whether the other PIPs have similar or divergent roles in petal development. The functions of NIPs during petal development is unclear; our results suggest that NIPs (c40917_g1, c45771_g1, c33177_g1) participate in petal expansion, but further research is needed to test this hypothesis. Petal protoplast observations showed that petal cells expand rapidly and undergo internal structural changes during flower opening. PE, PG, PL, and XYL may play important roles in the endomembrane system^29^, which suggests that this pathway also underwent transcriptional changes during petal development. We suggest that CES, XYL, PE, PG and PL could be involved in modifying the petal cell wall, changing turgor pressure, and remodelling the cytoskeleton. These downstream functional genes could be used as genetic markers to indicate important phenotypic changes and enable the development of an efficient screen for upstream regulators of these pathways.

The genes of eight phytohormone pathways were differentially expressed during the different stages of petal development. Although ABA is a natural regulator of petal senescence in flowers^30^,^31^, it may also play a role during petal development. Interestingly, most ABA signalling pathway members were down-regulated in FB_CP vs. FB_GP and up-regulated in OF_PP vs. FB_PP, highlighting a transcription level role for ABA in petal development that requires further research. ETH has a dose dependent effect in promoting or inhibiting flower opening, with cultivar specific differences in sensitivity^31^,^32^. The ETH pathway genes did not exhibit drastic expression changes in this study, which may be due to differences between the diploid rose ‘Old Blush’ and tetraploid cut rose. The unigene c35185_g1, has been previously registered in GenBank, and was found to be involved in the CK-regulated formation of rhizoids as a type-A response regulator in R. *canina*^33^, suggesting that this gene may have specific functions in different organs. The hormone biosynthesis or catabolism genes are not displayed in Fig. 8 because the petals may have been influenced by hormones produced by other flower organs; for example, the stamens of Arabidopsis produced high concentrations of AUX that inhibited petal growth during early flower development^34^, while the sepals may be a source of GA during flower bud development of *R. hybrida*^35^. There is a lot of crosstalk between phytohormone signals during petal development; for example, the AUX response and JA synthesis pathways have positive feedbacks that may ensure that the petals and stamens grow rapidly in Arabidopsis^36^. In *Gerbera hybrida*, GA and ABA regulate cell expansion during petal growth in an antagonistic manner^37^, while in *R. hybrida*, ABA and ETH promote petal senescence, but GA delays this process^9^. The reasons for these inconsistent results may be a concentration effect, inter-specific differences, organ or developmental differences, or may be because the treatments did not consider the influences of hormones from other organs.

The transcription factor expression levels change drastically during every developmental stage of the rose petals, indicating that, as a flower organ, their development may be controlled by a complex transcriptional regulation. As the richest groups of TFs in plants, the MYBs, are involved in plant development, secondary metabolism, hormone signal transduction, disease resistance, and abiotic stress tolerance^38^. Two previously identified MYBs, MYB12 and MYB113 (unigenes c30869_g1 and c33950_g1, respectively), may be involved in flavonol biosynthesis and anthocyanin biosynthesis, respectively^39^,^40^. Three MYB-like TFs (c46543_g4, c37323_g1 and c29224_g1) were annotated as the trichome differentiation protein GL1-like and should be further investigated, as they could potentially participate in the development of the rose petal conical epidermal cells^41^, further contributing to petal formation. AP2-like TFs function in the control of flower development^42^; therefore, unigenes c39981_g3, c39356_g1, and c43206_g2, which were annotated as AP2-like TFs, may be involved in rose petal development. Previously, it was reported that the target genes of DREB TFs are induced by abiotic stresses, while ERF dependent transcriptional regulation is linked to biotic stresses^43^,^44^. These TFs, which respond to stresses, may also regulate development. The AP2-EREBP TFs may integrate environmental signals, phytohormones, and rose petal development; however, further research is needed. The unigene c39802_g1 may be VIP1, which has been shown to be a regulator of osmosensory signalling and response in Arabidopsis, as well as a regulator of responses to mechanical stimuli^45^. Some of the differentially expressed TFs during petal development may participate in petal tissue differentiation and expansion, such as SBP and C3H-WRC/GRF. SBP was previously suggested to be involved in flower development and differentiation^46^ and C3H-WRC/GRF may regulate the growth and shape of leaves and petals^47^. By contrast, the functions of other TFs in petal development are unclear, such as BTB-POZ, which may perform crucial roles in both male and female gametophyte development^48^. For some of these transcription factors, such as those of the MYB, AP2-EREBP, NAC and bZIP families, we can presume their function and putative roles in petal development; however, some novel TFs may also play important and as yet undetermined roles in rose petal development, such as SBP, C2H2 and C3H-WRC/GRF TFs. The TFs in our database can be evaluated as genetic linkers between the upstream signalling and downstream functional genes in the regulation of rose petal development.

In summary, we used RNA-seq technology to provide an overview of the dynamic transcriptome changes during rose petal development. Through pairwise comparison analyses of DEGs between each petal developmental stage, we identified many transcripts likely to participate in flower colouring and petal expansion, and many novel transcripts that may be involved in petal growth and development. In addition, we assessed expression differences in the phytohormone pathways and determined transcription factors with highly variable expression between the different stages. Overall, the resources generated in this study can be used to facilitate the further dissection of the molecular mechanisms underlying rose petal development and improve the efficiency of ornamental plant molecular breeding.

## Material and Methods

### Plant materials

Petals of *Rosa chinensis* ‘Old Blush’ were collected from plants grown in the greenhouse, under a 12 h light (6:00 am–18: 00 pm)/12 h dark (18:00 pm–6:00 am) photoperiod and a 25 °C day/18 °C night temperature regime. In order to maintain the plant material consistency, we collected petals at 12:00 am under 25 °C. Based on the observed changes in petal phenotype during development, the process was divided into four typical stages: green petals in the flower bud (FB_GP), colour changing petals in the flower bud (FB_CP), pink petals in the flower bud (FB_PP), and pink petals of the open flower (OF_PP). After flower bud formation and the elongation of the stalk to 3 cm, the flower bud gradually dilated and contained green petals (FB_GP). After two days, the petals’ colour started to change to pink, and the flower bud gradually expanded (FB_CP). After three days, the flower bud stopped growing and the sepals cracked slightly, revealing the now dark pink petals within (FB_PP). About two days later, the flower was fully open, and the petals had expanded to their maximum size and retained a pink colour (OF_PP). The petals of six flowers at each developmental stage were collected as one sample and immediately frozen in liquid nitrogen. Two biological replicates were performed for each stage, for a total of eight samples.

### Determination of anthocyanin content

For each sample, 0.1 g of frozen material was mixed with 2 mL 0.1% HCl in methanol and homogenised for 30 min using ultrasonic vibration in ice water. The samples were centrifuged for 10 min at 13,000 g and the resulting supernatant was passed through a 0.22 μm filter. The supernatant of stage FB_PP, which had the most pigmented petals, was analysed for the presence of different anthocyanins by liquid chromatography/mass spectrometry using an ion trap-time off light system (LCMS-IT-TOF^TM^, Shimadzu, Kyoto, Japan), which was coupled to a diode-array detection system. The mass spectrometer was operated in positive ion mode. The anthocyanin content of each sample was quantified by high performance liquid chromatography (Alliance Separations Module 2695, Waters, MA, USA) using a 4.6 × 150 mm C18 column, which was maintained at a temperature of 25 °C. The flow rate was 0.8 mL · min^−1^ and the sample volume was 10 μL. The mobile phase consisted of (A) 0.5% methanoicacid and (B) acetonitrile, with the following gradient: 0 min: 5% B; 5 min: 10% B; 30 min: 19% B; 55 min: 40% B; 55.01–60 min: 5% B. The detection wavelength was 520 nm. The anthocyanin standards Cy-3,5-diglucoside and Cy-3-glucoside were purchased from Sigma-Aldrich (MO, USA). These experiments were performed three times.

### Isolation and measurement of petal protoplasts

The protoplasts of petal samples from three developmental stages (FB_GP, FB_PP, and OF_PP) were isolated as previously described^49^. These protoplasts were photographed and measured using Zeiss Axio Scope A1, following the manufacturer’s recommendations. A total of 20 high quality protoplasts were imaged from each stage, and three biological repeats were performed.

### RNA preparation, cDNA library construction, and RNA-seq

Petal RNA was extracted using an SV Total RNA Isolation Kit (Promega, WI, USA), according to the manufacturer’s instructions. RNA degradation and contamination were examined on a 1% agarose gel. A Nano Photometer^®^ spectrophotometer (IMPLEN, CA, USA) and an Agilent Bioanalyzer 2100 system RNA Nano 6000 Assay Kit (Agilent Technologies, CA, USA) were used for checking RNA purity and integrity. The concentration of the extracted RNA was measured using a Qubit^®^ RNA Assay Kit with a Qubit^®^ 2.0 Fluorometer (Life Technologies, CA, USA).

A total of 3 μg RNA for each sample was prepared for sequencing as follows. A NEBNext^®^ UltraTM RNA Library Prep Kit (New England Biolabs, MA, USA) was used to generate libraries for next-generation sequencing on the Illumina^®^ platform (Illumina, CA, USA), following the manufacturer’s recommendations. Poly-T oligo-attached magnetic beads were used to isolate mRNA from the total RNA. First strand cDNA was synthesised using random hexamer primers and a M-MuLV Reverse Transcriptase (RNase H-; New England Biolabs), with DNA polymerase I and RNase H used to perform the subsequent second strand cDNA synthesis. NEBNext Adaptors (New England Biolabs) were ligated to the cDNA libraries to prepare for hybridisation after the adenylation of 3′ ends of the cDNA fragments. The library fragments were purified using the Agencourt AMPure XP system (Beckman Coulter, CA, USA) and 150–200 bp cDNA fragments were selected. The adaptor-ligated and size-selected cDNA was mixed with 3 μL USER Enzyme (New England Biosystems) and incubated at 37 °C for 15 min and then at 95 °C for 5 min. Multiplex PCR was performed using Phusion High-Fidelity DNA polymerase, universal PCR primers, and the NEBNext Index (X) Primer (New England Biosystems), and following these steps: 1) 98 °C for 10 s; 2) 98 °C for 10 s, 65 °C for 30 s, 72 °C for 30 s, repeats 15 cycles; 3) 72 °C for 5 min. An Agilent Bioanalyzer 2100 system (Agilent Technologies) was used to assess the library quality. Using a TruSeq PE Cluster Kit v3-cBot-HS (Illumina), the index-coded samples were clustered on a cBot Cluster Generation System (Illumina), according to the manufacturer’s instructions. The library was sequenced using an Illumina HiSeq™PE125 to generate paired end reads. All raw-sequence reads data were submitted as BioProject (PRJNA351281) to NCBI Sequence Read Archive (SRA, http://www.ncbi.nlm.nih.gov/Traces/sra) with accession number SRP092271.

### Quality control, transcriptome assembly, and gene functional annotation

Reads containing adapter, poly-N, or low quality sequences were removed from the analysis. Clean reads were determined by their error rate, Q20, Q30, and GC-contents, and the analyses were performed on clean reads with high quality sequences. Transcriptome assembly was accomplished using Trinity^11^ with min_kmer_cov set to 2 by default and all other parameters set as default. Seven databases were used to annotate the gene function: Nr (NCBI non-redundant protein sequences, NCBI blast 2.2.28+, e-value = 1e^−5^); Nt (NCBI non-redundant nucleotide sequences, NCBI blast 2.2.28+, e-value = 1e^−5^); Pfam (Protein family, http://pfam.sanger.ac.uk/, HMMER 3.0 package, hmmscan, e-value = 0.01); KOG/COG (Clusters of Orthologous Groups of proteins, http://www.ncbi.nlm.nih.gov/COG/, NCBI blast 2.2.28+, e-value = 1e^−3^); Swiss Prot (a manually annotated and reviewed protein sequence database, http://www.ebi.ac.uk/uniprot/, NCBI blast 2.2.28+, e-value = 1e^−5^); KO (KEGG Ortholog database, http://www.genome.jp/kegg/, KAAS, KEGG Automatic Annotation Server, e-value = 1e^−10^)^50^; and GO (Gene Ontology, http://www.geneontology.org/, Blast2GO v2.5 (e-value = 1e^−6^)^51^.

### Differential gene expression analysis

For each sample, the gene expression levels were estimated by RSEM (bowtie2 default parameters, mismatch = 0)^52^. Transcripts with FPKM values of >1 in at least one sample were defined as the threshold expression of that gene. A differential expression analysis between the transcriptomes of different developmental stages (FB_CP vs. FB_GP, FB_PP vs. FB_CP, OF_PP vs. FB_PP) was performed using the DESeq R package (version 1.10.1). The Benjamini-Hochberg procedure was used to adjust the resulting P-values to control for the false discovery rate (FDR < 0.001)^53^. Any gene with an adjusted P-value of <0.05 was determined to be differentially expressed. Heat maps of gene expression were produced using HemI software^54^ based on the log2.Fold_change value of FB_CP vs. FB_GP, FB_PP vs. FB_CP, and OF_PP vs. FB_PP. The seven database annotations were used to identify genes involved in the anthocyanin pathway, petal expansion, and phytohormone pathways, as well as the transcription factors. The identified candidate transcription factors were validated in PlantTFcat (http://plantgrn.noble.org/PlantTFcat/)^23^.

### Gene Ontology and KEGG Ortholog enrichment analysis

The GO seq R packages, which can adjust for gene length bias in DEGs, is based on Wallenius’ non-central hyper-geometric distribution^55^. It was used to implement the GO-enrichment analysis of DEGs from the petal transcripts, as well as the up- and down-regulated DEGs (adjusted P-value < 0.05) from the three libraries (FB_CP vs. FB_GP, FB_PP vs. FB_CP, OF_PP vs. FB_PP). KOBAS software (KEGG Orthology-Based Annotation System)^56^ was used to test the statistical enrichment of DEGs in KEGG pathways (adjusted P-value < 0.05).

### qPCR validation

The total RNA of petals was extracted using an SV Total RNA Isolation System (Promega), according to the manufacturer’s instructions. First-strand cDNA was synthesised from the total RNA using PrimeScript^TM^ RT Reagent Kit with gDNA Eraser (Takara Bio Inc., Shiga, Japan), following the manufacturer’s instructions. The qPCR was performed using the following parameters: initial denaturation for 30 s at 95 °C, followed by 40 cycles of 5 s at 95 °C and 30 s at 60 °C, concluding with a melting-curve stage for 15 s at 95 °C, 1 min at 60 °C, and 15 s at 95 °C. Each reaction consisted of 2 μL first-strand cDNA, 10 μL SYBR Premix Ex Taq (Takara), 0.4 μLeach of 10 μM upper and lower primers and 7.2 μL sterile distilled water (dH_2_O). Each sample was assessed in three technical replicates for each of three biological repeats and normalised using RcACTIN as an internal control. The transcription levels are presented in the form 2^−ΔΔCT^ 55. The scatter diagrams were generated using Origin8 software (OriginLab, Northampton, MA, USA).

## Additional Information

**How to cite this article:** Han, Y. *et al*. Comparative RNA-seq analysis of transcriptome dynamics during petal development in *Rosa chinensis. Sci. Rep.* **7**, 43382; doi: 10.1038/srep43382 (2017).

**Publisher's note:** Springer Nature remains neutral with regard to jurisdictional claims in published maps and institutional affiliations.

## Supplementary Material

Supplementary Datasets

Supplementary Table S 9

Supplementary Table S 10

Supplementary Table S 11

Supplementary Table S 12

Supplementary Table S 13
