# Supplementary material for: Comparative RNA-seq analysis of transcriptome dynamics during petal development in Rosa chinensis
Source: Sci Rep. 2017 Feb 22;7:43382. doi: 10.1038/srep43382 (PMC5320579; doi:10.1038/srep43382)
Supplement: Supplementary Datasets [file srep43382-s1.pdf]

Title

Comparative RNA-seq analysis of transcriptome dynamics during petal development in *Rosa chinensis*

Author list

Yu Han[1], Huihua Wan[1], Tangren Cheng[1], Jia Wang[1], Weiru Yang[1], Huitang Pan[1*]& Qixiang Zhang[1*]

Supplementary Information

Supplementary Figure S1. Calibration curve of Cy-3,5-diglucoside in HPLC standards.

Supplementary Figure S2. Distribution of transcript read lengths.

Supplementary Figure S3. Pearson correlation between two biological replicates in the four developmental stage samples.

Supplementary Figure S4. Species from which the transcript annotation was derived in the Nr database.

Supplementary Figure S5. GO enrichment analysis of up- and down-regulated differentially expressed genes in FB_CP vs. FB_GP, FB_PP vs. FB_CP and OF_PP vs. FB_PP.

Supplementary Figure S6. qPCR validation of transcript levels evaluated by RNA-seq.

Supplementary Table S1. The quality analysis of all reads from eight samples.

Supplementary Table S2. Length frequency distribution of assembly transcripts and unigenes.

Supplementary Table S3. Length distribution of assembly transcripts and unigenes.

Supplementary Table S4. Success rate statistics of transcript annotation using seven databases.

Supplementary Table S5. GO annotation classification frequencies.

Supplementary Table S6. KOG annotation classification frequencies.

Supplementary Table S7. KO annotation classification frequencies.

Supplementary Table S8. Number of total clean reads of the eight samples mapped to reference sequences.

Supplementary Table S9. FPKM (fragments per kilobase of transcript per million base pairs sequenced) of the four developmental stage samples (FB_GP, FB_CP, FB_PP, OF_PP).

Supplementary Table S10. Up- and down-regulated DEGs in the transcriptome comparisons of FB_CP vs. FB_GP, FB_PP vs. FB_CP, and OF_PP vs. FB_PP.

Supplementary Table S11. Differentially expressed genes of the anthocyanin and petal expansion regulatory pathways.

Supplementary Table S12. Differentially expressed genes of the phytohormone biosynthesis and signalling pathways.

Supplementary Table S13. Transcription factors with ≥ five-fold change in expression during rose petal development.

Supplementary Table S14. Upper and lower primer sequences of genes for qPCR experiments.

Supplementary Figure S1. Calibration curve of Cy-3,5-diglucoside in HPLC standards.
Y (content) = $4.96\times10^{7}\times$X (area) - 7.98×10^{4}, R^2 = 0.999727, Standard error = 1.603642×10^{5}

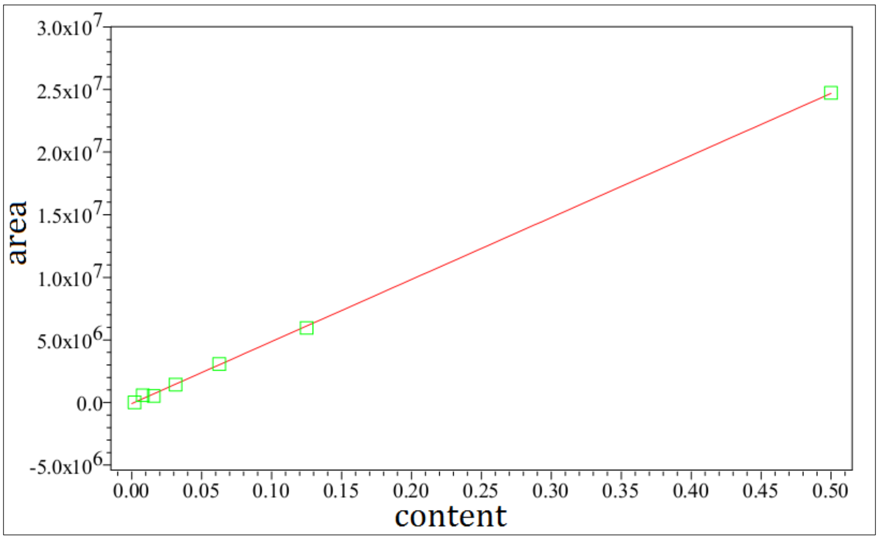

Supplementary Figure S2. Distribution of transcript read lengths in eight samples. The x-axis denotes the distance from the 5’ end of transcript (%), while the y-axis shows the average distribution of reads.

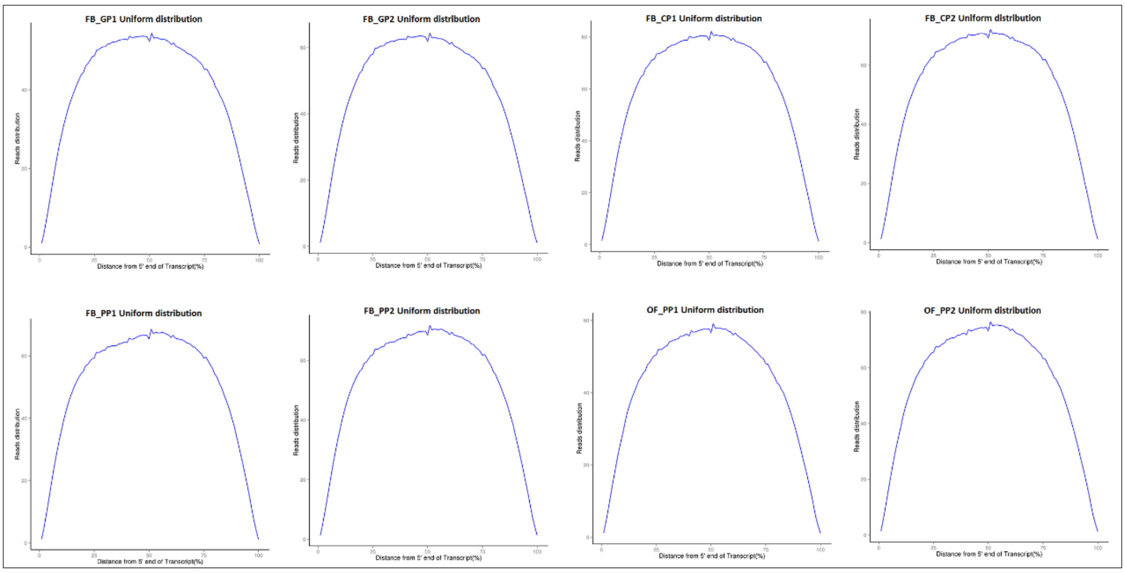

Supplementary Figure S3. Pearson correlation between two biological replicates in the four developmental stage samples.

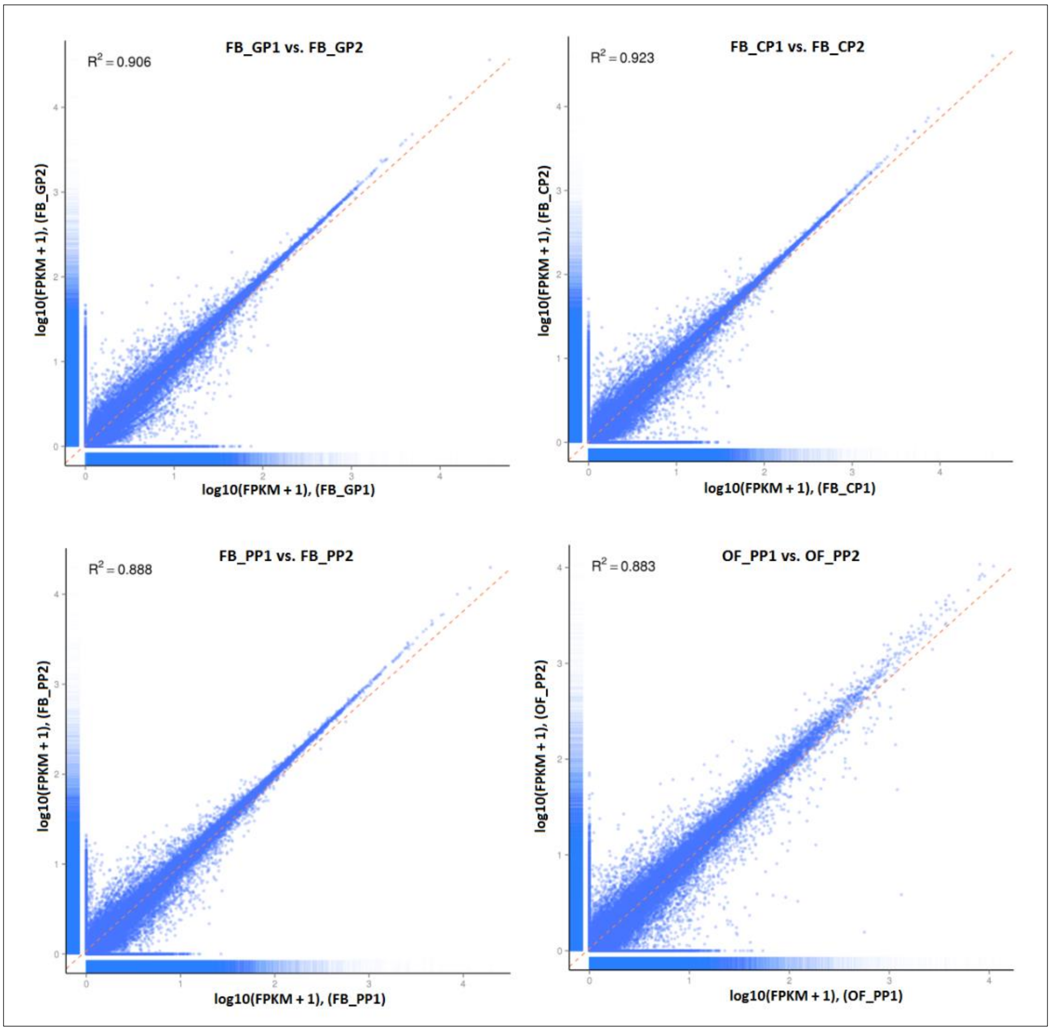

Supplementary Figure S4. Species from which the transcript annotation was derived in the Nr database.

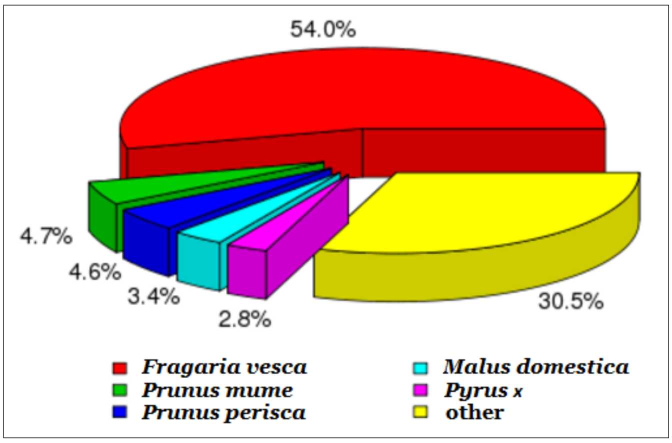

Supplementary Figure S5. GO significant enrichment analysis of up- and down-regulated differentially expressed genes in FB_CP vs. FB_GP, FB_PP vs. FB_CP and OF_PP vs. FB_PP.

GO contents three term types: Biological process (red bar), Cellular component (blue bar), and Molecular function (green bar). Adjusted P-Value < 0.05.

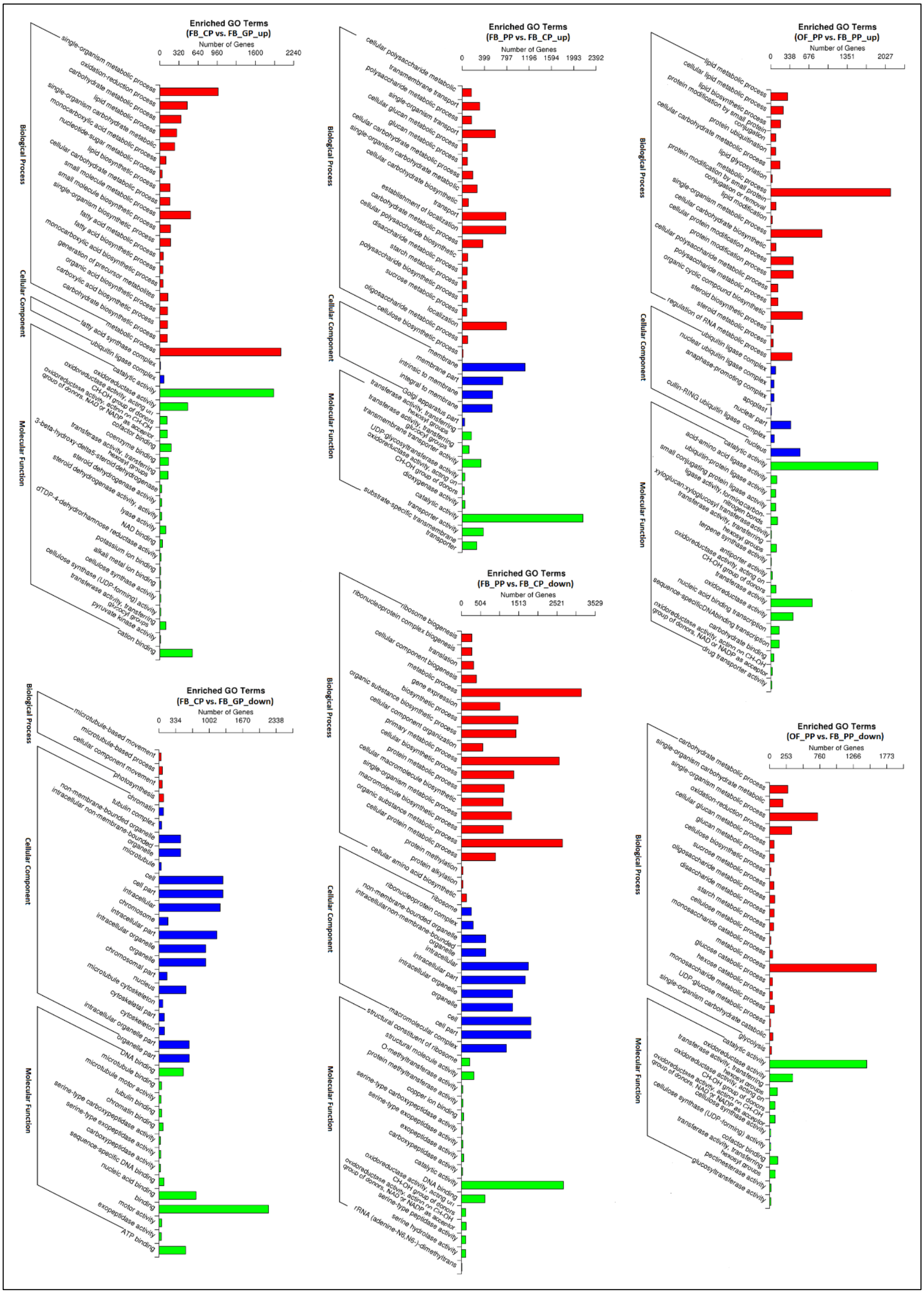

Supplementary Figure S6. qPCR validation of transcript levels evaluated by RNA-seq. (A-C) Correlation of the fold change analysed by RNA-seq platform with data obtained using qPCR in FB_CP vs. FB_GP, FB_PP vs. FB_CP, and OF_PP vs. FB_PP, respectively. The information for each gene is shown in Supplementary Table S14. All qPCR data were collected from three biological replicates and there were three technical replicates for each sample. All standard errors within reasonable ranges.

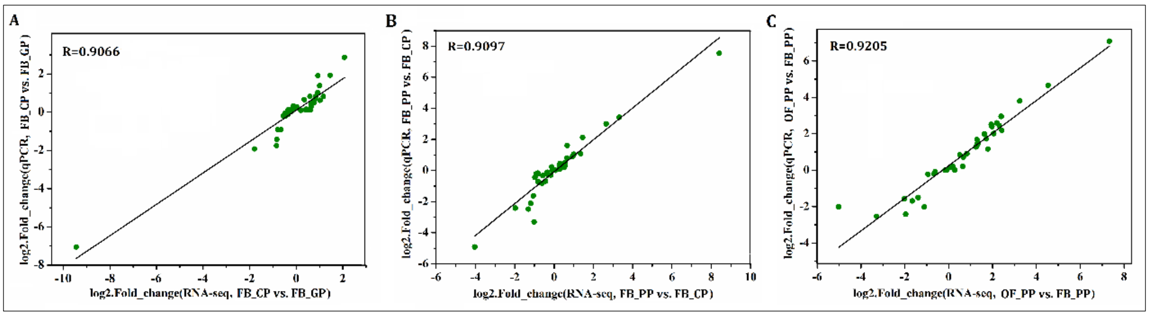

Supplementary Table S1.The quality analysis of all reads from eight samples. Error (%) represent the error rate of sequenced bases. The error rate of all samples are not more than 0.02%.

Sample	Raw reads	Clean reads	Clean bases	Error (%)	Q20 (%)	Q30 (%)	GC (%)
FB_GP1	55484658	54644178	8.20Gbp	0.02	96.73	92.23	46.10
FB_GP2	67450752	66371336	9.96Gbp	0.01	96.89	92.59	46.05
FB_CP1	88489712	86996010	13.05Gbp	0.01	97.03	92.94	45.89
FB_CP2	76817970	75621484	11.34Gbp	0.01	96.99	92.82	45.87
FB_PP1	80178264	77216966	11.58Gbp	0.02	96.80	92.35	45.47
FB_PP2	88366662	86011680	12.90Gbp	0.01	97.09	93.09	45.48
OF_PP1	69454680	67793180	10.17Gbp	0.01	97.01	92.85	45.73
OF_PP2	85285886	83457910	12.52Gbp	0.01	97.14	93.12	45.77
Total	611528584	598112744	89.72Gbp				

Supplementary Table S2. Length frequency distribution of assembly transcripts and unigenes.

Transcript length interval	200-500bp	500-1Kbp	1K-2Kbp	>2Kbp	Total
Number of transcripts	89937	40130	41206	36766	208039
Number of unigenes	70676	18686	12205	10387	111954

Supplementary Table S3. Length distribution of assembly transcripts and unigenes. N50 and N90 are defined as follows: the assembly transcripts are ordered from longest to shortest, and N50 is the length of transcripts that are no less than 50% the total length, N90 is the length of transcripts that are no less than 90% the total length.

Assembly type	Min length	Mean length	Median length	Max length	N50	N90	Total nucleotides
Transcripts	201	1111	631	15940	1997	438	231028056
Unigenes	201	751	360	15940	1467	270	84096145

Supplementary Table S4. Number of total clean reads of the eight samples mapped to reference sequences.

Sample name	Total reads	Total mapped
FB_GP1	54644178	42539318 (77.85%)
FB_GP2	66371336	51522634 (77.63%)
FB_CP1	86996010	67977742 (78.14%)
FB_CP2	75621484	58940310 (77.94%)
FB_PP1	77216966	60096450 (77.83%)
FB_PP2	86011680	66360294 (77.15%)
OF_PP1	67793180	52792804 (77.87%)
OF_PP2	83457910	65651108(78.66%)

Supplementary Table S5. Success rate statistics of transcript annotation using seven databases. Nr - NCBI non-redundant protein sequences; Nt - NCBI non-redundant nucleotide sequences; KO - KEGG Ortholog database; SwissProt - A manually annotated and reviewed protein sequence database; Pfam - Protein family; GO - Gene Ontology; KOG - Clusters of Orthologous Groups of proteins.

Annotation type	Number of unigenes	Percentage (%)
Annotated in Nr	45138	40.31
Annotated in Nt	41005	36.62
Annotated in KO	19605	17.51
Annotated in SwissProt	37690	33.66
Annotated in Pfam	35529	31.73
Annotated in GO	35830	32.00
Annotated in KOG	19897	17.77
Annotated in at least one database	61456	54.89
Total unigenes	111954	

Supplementary Table S6. GO annotation classification frequencies.

GO ID	GO term	GO term	Gene number
GO:0000003	Reproduction	Biological process	240
GO:0023052	Signaling	Biological process	2601
GO:0051704	Multi-organism process	Biological process	1799
GO:0048519	Negative regulation of biological process	Biological process	281
GO:0048511	Rhythmic process	Biological process	17
GO:0001906	Cell killing	Biological process	29
GO:0040007	Growth	Biological process	42
GO:0032502	Developmental process	Biological process	501
GO:0022414	Reproductive process	Biological process	268
GO:0022610	Biological adhesion	Biological process	320
GO:0009987	Cellular process	Biological process	19558
GO:0044699	Single-organism process	Biological process	14661
GO:0040011	Locomotion	Biological process	317

GO:0044848	Biological phase	Biological process	13
GO:0065007	Biological regulation	Biological process	6564
GO:0050896	Response to stimulus	Biological process	4315
GO:0008152	Metabolic process	Biological process	18860
GO:0032501	Multicellular organismal process	Biological process	706
GO:0050789	Regulation of biological process	Biological process	6129
GO:0071840	Cellular component organization or biogenesis	Biological process	3288
GO:0048518	Positive regulation of biological process	Biological process	340
GO:0002376	Immune system process	Biological process	156
GO:0051179	Localization	Biological process	5502
GO:0030054	Cell junction	Cellular component	43
GO:0005576	Extracellular region	Cellular component	701
GO:0044425	Membrane part	Cellular component	5163
GO:0031012	Extracellular matrix	Cellular component	254
GO:0019012	Virion	Cellular component	852
GO:0032991	Macromolecular complex	Cellular component	7399
GO:0016020	Membrane	Cellular component	5533
GO:0044464	Cell part	Cellular component	11078
GO:0044423	Virion part	Cellular component	852
GO:0043226	Organelle	Cellular component	7354
GO:0009295	Nucleoid	Cellular component	5
GO:0044420	Extracellular matrix part	Cellular component	114
GO:0045202	Synapse	Cellular component	6
GO:0031974	Membrane-enclosed lumen	Cellular component	969
GO:0044421	Extracellular region part	Cellular component	668
GO:0005623	Cell	Cellular component	11085
GO:0044456	Synapse part	Cellular component	6
GO:0044422	Organelle part	Cellular component	3975
GO:0005215	Transporter activity	Molecular function	2387
GO:0030234	Enzyme regulator activity	Molecular function	615
GO:0004872	Receptor activity	Molecular function	355
GO:0005198	Structural molecule activity	Molecular function	1326
GO:0005488	Binding	Molecular function	20313
GO:0000988	Protein binding transcription factor activity	Molecular function	332
GO:0060089	Molecular transducer activity	Molecular function	615
GO:0001071	Nucleic acid binding transcription factor activity	Molecular function	1017
GO:0030545	Receptor regulator activity	Molecular function	21
GO:0016530	Metallochaperone activity	Molecular function	13
GO:0003824	Catalytic activity	Molecular function	15753
GO:0016209	Antioxidant activity	Molecular function	199
GO:0005085	Guanyl-nucleotide exchange factor activity	Molecular function	49
GO:0016247	Channel regulator activity	Molecular function	146
Total annotated genes			35830

Supplementary Table S7. KOG annotation classification frequencies.

KOG term	Gene number
RNA processing and modification	1072
Chromatin structure and dynamics	340
Energy production and conversion	1087
Cell cycle control, cell division, chromosome partitioning	566
Amino acid transport and metabolism	847
Nucleotide transport and metabolism	267
Carbohydrate transport and metabolism	932
Coenzyme transport and metabolism	211
Lipid transport and metabolism	823
Translation, ribosomal structure and biogenesis	1751
Transcription	1016
Replication, recombination and repair	461
Cell wall/membrane/envelope biogenesis	200
Cell motility	20
Posttranslational modification, protein turnover, chaperones	2670
Inorganic ion transport and metabolism	579
Secondary metabolites biosynthesis, transport and catabolism	756
General function prediction only	3389
Function unknown	737
Signal transduction mechanisms	2090
Intracellular trafficking, secretion, and vesicular transport	1200
Defense mechanisms	161
Extracellular structures	128
Unnamed protein	3
Nuclear structure	113
Cytoskeleton	928
Total annotated genes	19897

Supplementary Table S8. KO annotation classification frequencies.

KO pathway hierarchy1	Pathway hierarchy2	Gene number
Cellular processes	Cell growth and death	751
Cellular processes	Cell motility	317
Cellular processes	Cellular community	696
Cellular processes	Transport and catabolism	1224
Environmental information processing	Membrane transport	119
Environmental information processing	Signal transduction	2229
Environmental information processing	Signaling molecules and interaction	208
Genetic information processing	Folding, sorting and degradation	1557
Genetic information processing	Replication and repair	313
Genetic information processing	Transcription	804
Genetic information processing	Translation	2040
Metabolism	Amino acid metabolism	1082

Metabolism	Biosynthesis of other secondary metabolites	345
Metabolism	Carbohydrate metabolism	1605
Metabolism	Energy metabolism	1321
Metabolism	Glycan biosynthesis and metabolism	304
Metabolism	Lipid metabolism	799
Metabolism	Metabolism of cofactors and vitamins	472
Metabolism	Metabolism of other amino acids	465
Metabolism	Metabolism of terpenoids and polyketides	290
Metabolism	Nucleotide metabolism	490
Metabolism	Overview	1175
Metabolism	Xenobiotics biodegradation and metabolism	246
Organismal systems	Circulatory system	447
Organismal systems	Development	211
Organismal systems	Digestive system	408
Organismal systems	Endocrine system	1124
Organismal systems	Environmental adaptation	580
Organismal systems	Excretory system	312
Organismal systems	Immune system	879
Organismal systems	Nervous system	711
Organismal systems	Sensory system	149
Total annotated genes		19605

Supplementary Table S9. FPKM (fragments per kilobase of transcript per million base pairs sequenced) of the four developmental stage samples (FB_GP, FB_CP, FB_PP, OF_PP).

In the individual excel format “Supplementary Table S9”.

Supplementary Table S10. Up- and down-regulated DEGs in the transcriptomic comparisons of FB_CP vs. FB_GP, FB_PP vs. FB_CP, and OF_PP vs. FB_PP. Gene ID is the sub-component serial number spliced by Trinity, p_{adj} (the adjusted *p* value) < 0.05.

In the individual excel format “Supplementary Table S10”.

Supplementary Table S11. Differentially expressed genes of the anthocyanin and petal expansion pathways.

In the individual excel format “Supplementary Table S11”.

Supplementary Table S12. Differentially expressed genes of the phytohormone biosynthesis and signalling pathways.

In the individual excel format “Supplementary Table S12”.

Supplementary Table S13. Transcription factors with ≥ five-fold change in expression during rose petal development.

In the individual excel format “Supplementary Table S13”.

Supplementary Table S14. Upper and lower primer sequences of genes evaluated using qPCR experiments.

Gene ID	Upper primers	Lower primers
PAL c45933_g1	TAACGGAGAGACCCTAACCC	AGAAGCCATACCAGACCCA
PAL c45930_g1	TGAGGCATTTGGAGGAGAA	CCCTTTCAACCACCATAAGC
4CL c27001_g1	TTCGTCAGGGACTACTGGTG	GTGAAACAATGGCAGAACAGAT
4CL c44050_g1	TTTGAGATTGGGACGCTTT	CGCCACCATAGGATTCTTC
4CL c44050_g2	GCAGGTTGACGGAGAGAATC	CCACATAGCAAGACCGAGTTC
4CL c44050_g3	TCCACATCTACTCCCTCAACTC	TCCACCAACTCCAACAACTT
4CL c39562_g1	TGTGCTCTTCCCTTCTCGT	TTCCTATCATTTCTGGCGTTAC
C4H c39153_g1	TAATGGGTCTCTTCGTCGC	TGTCAGATTGCGGTGGTT
CHS c40105_g3	TGCTACGGATTGCTAAAGACG	TGGCTGTCACAAGGTTGCT
F3H c44596_g1	TCGTCTCCAGCCATTTACAG	GGTCACATCTCTCCATCCCT
ANS c38343_g1	TAGAAGAAGGGAGGCTGGAG	GTGGAGGATGAAGGTGAGTG
LAR c42630_g2	CTTCTATTGCCTCTGCTGCTAA	CGCCTCTGTCTTTGAACGA
FLS c37896_g1	AAGCCACACAACATCATCGT	GCCAACCTTCTGTAACTCCTCT
F3'H c39849_g2	TGGTGTTGGCAGGAGTGTT	GCTTCTTCATCTTGGACGC
DFR c19467_g1	TCAAAGACACTGGCAGAGAAAG	CAGGCGTGAATGATGGAAG
DFR c37847_g1	TCAGGGAGACGAAACCGTAG	AAGATGGATGGACGCACCT
DFR c38644_g1	GTGGAAGAGAACATTGAGGCTT	CACAGGATTTGAGGAGGTCC
DFR c42488_g1	CGGAGAGTGAAGATGACTGG	TGGACCGATTACGAGAGTTG
3GT c48997_g1	AAATCCTCAATCATACCTCG	TTCCATACAACTTCTACACTCCG
PYR c30786_g1	GCACGGTCAGAGAAGGGTTT	TCATCGTCCAGCAAGTCCA
PYL c27077_g1	CCATAGCCAAACCATAGACG	TTGATGAACTGCTTGTGGG
PYL c45749_g1	TGGTCGTTAGTCAGGAGATTTG	CCAAGAATGTGCTCGTCGT
PYL c34567_g1	ACCAGAACCAGAAGGGCAT	TTGACGAAGTGCTTGTAGGC
PP2C c43068_g1	AAGGCACACATTCAAGCAAC	TGGACAAACAACGACGACTAC
PP2C c44063_g1	GAGAGGGAGGCAAAGTCATAA	GTCCTGGCTGTGAATGTAATCT
PP2C c44529_g1	CCAACAATCTTCACTACTACGCC	TCTCCTCCTTCACCACCTCA
PP2C c44889_g1	CCTGTTTCGGAGTTACCTGG	CTTCGGATGGCTGCTTTAC
PP2C c45167_g1	TGGCGGAGGAGATAGAACTT	TCCAACTTCAGCATCAACCT
PP2C c46051_g1	TTTGCGGAAGGAGAAGAGA	CGTCGTAAACACCGTAGAA
PP2C c45180_g2	GCAGCAGAAAGCGAATGAG	AACGACGACGAGAACGACA
SnRK2 c37054_g1	GGACCAACAATCTCAACCAGT	GCTGCTGTCAATGTCAAGGT
SnRK2 c39827_g1	GCAGATTTGATGGACGAGATG	TACGGGCTAAGATTGTGGATTC
SnRK2 c40929_g1	ACCACCCAACACAGAGCAT	CAAGCAAAGTCACCACTGAGA
SnRK2 c42473_g1	GACACCAACTCATTTAGCCATT	AAACCTTGCCTCATTCTCACTA
SnRK2 c45989_g3	TCACTTCGCCATCCAAAC	GCGGCATACTCCATAACAATAG
SnRK2 c47011_g2	TGGAACTGATGGAAGGAGG	GATGAGATGCGTAACTGCGT
ABF c41799_g1	GAGAGAAGGCAAAGGAGAATG	GGATGGCAGAAGTAATGTGG
ABF c47050_g1	TTGGGATTCGGGTTTCAG	CATTAGCATTCAGTGGCAGATT
RcACTIN	CAATGCTCCCGCTATGTATG	AGGTCAAGTCGCAGAATGG
